# Supplementary material for: The AE4 transporter mediates kidney acid-base sensing
Source: Nat Commun. 2023 May 26;14:3051. doi: 10.1038/s41467-023-38562-x (PMC10220024; doi:10.1038/s41467-023-38562-x)
Supplement: Supplementary file 1 — Supplementary Information [file 41467_2023_38562_MOESM1_ESM.pdf]

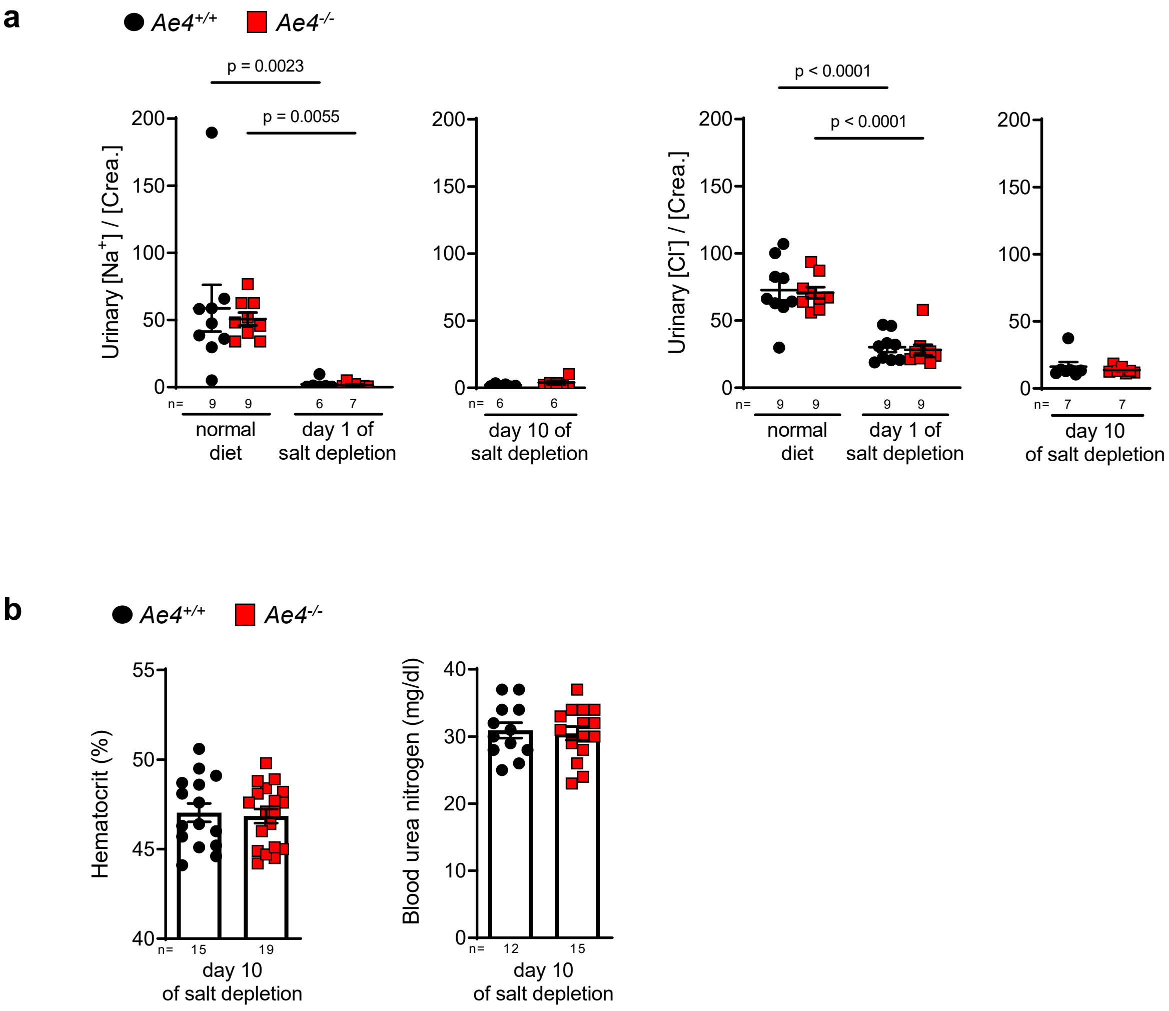

Supplementary Fig. 1 AE4-deficient mice display a normal renal response to salt depletion. *Ae4*$^{-/-}$ and wildtype (*Ae4*$^{+/+}$) mice were fed a normal diet or challenged for 10 days with a sodium-depleted diet. **a** Urinary Na^+ and Cl^- excretion were equally reduced in both genotypes during NaCl-depleted diet. Urinary electrolyte excretion was measured 20 hours after onset of diet (n=6-9 animals per genotype, two-way-ANOVA followed by Bonferroni's multiple comparisons test) and after 10 days of diet (n=6-7 animals per genotype, two-tailed Student’s t-test). **b** Hematocrit, and BUN were not different between *Ae4*$^{+/+}$ and *Ae4*$^{-/-}$ after 10 days of NaCl-depleted diet (n=12-19 animals per genotype, two-tailed Student’s t-test). All data are presented as mean ± SEM and each point or square denotes one animal. Source data are provided as a Source Data file.

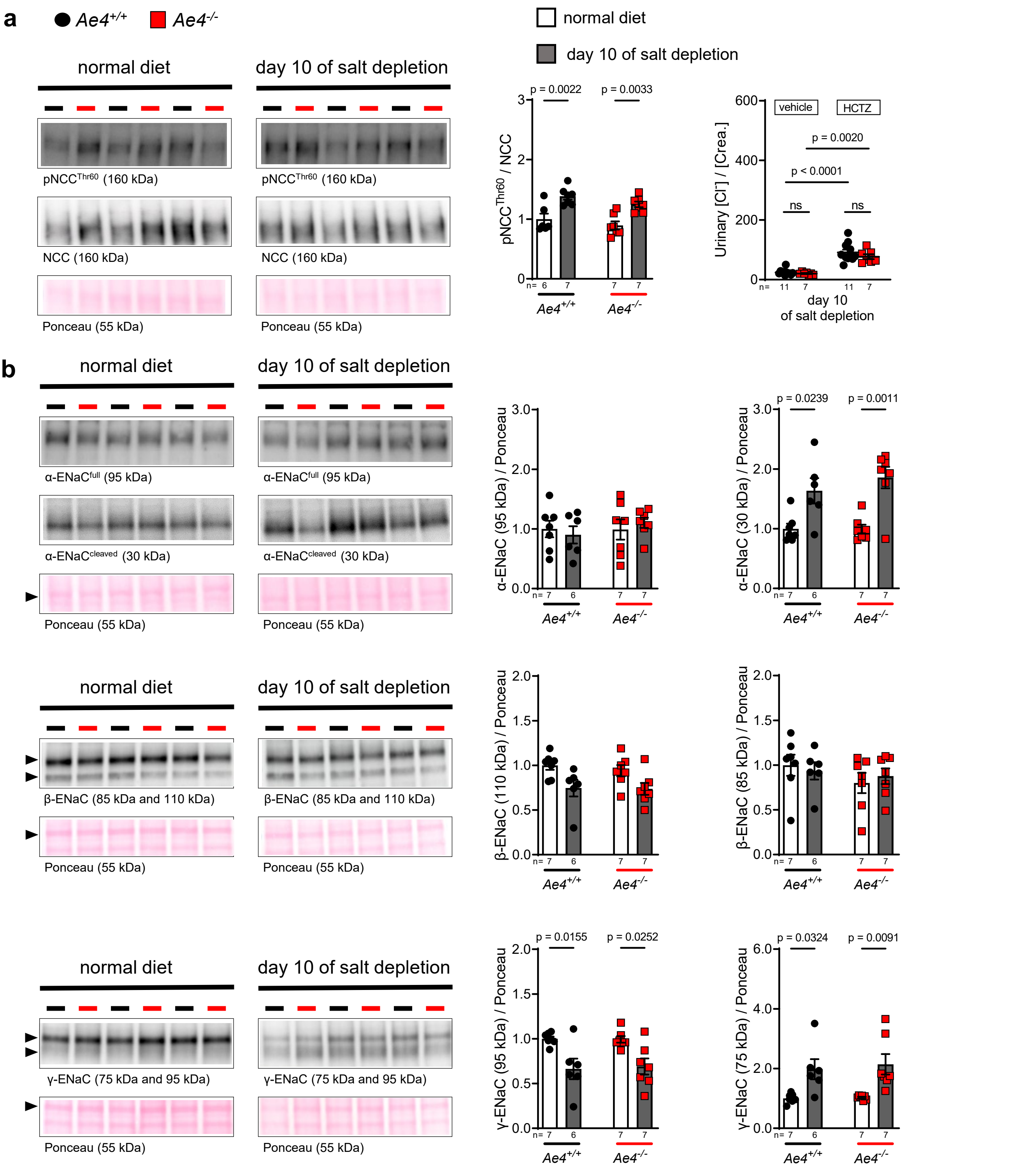

Supplementary Fig. 2 AE4-deficient mice display a normal activation of renal salt reabsorption pathways in response to salt depletion. *Ae4*$^{-/-}$ and wildtype (*Ae4*$^{+/+}$) mice were fed a normal diet or challenged for 10 days with a sodium-depleted diet. **a** Representative immunoblots of total renal sodium/chloride cotransporter (NCC) and pNCC protein levels, ponceau red staining indicates equal loading. Graphs exhibit densitometric quantification of protein levels normalized to ponceau, relative levels to *Ae4*$^{+/+}$ are plotted (n=6-7 animals per genotype, one-way ANOVA followed by Bonferroni's multiple comparison test). The effect of the NCC blocker hydrochlorothiazide (HCTZ, 10 mg/kg) on urinary Cl$^-$ excretion was not different (ns: not siginifcant p>0.05) between both genotypes (n=7-11 animals per genotype, two-way ANOVA followed by Bonferroni's multiple comparisons test). **b** Representative immunoblots of full α- (95 kDa), cleaved α- (30 kDa), β-, full γ- (95 kDa), and cleaved γ- (75 kDa) ENaC subunits in *Ae4*$^{+/+}$ and *Ae4*$^{-/-}$ kidneys are shown. Graphs exhibit densitometric quantification of protein levels normalized to ponceau, relative levels to *Ae4*$^{+/+}$ are plotted (n=6-7 animals per genotype, one-way ANOVA followed by Bonferroni's multiple comparison test). All data are presented as mean ± SEM and each point or square denotes one animal. Source data are provided as a Source Data file.

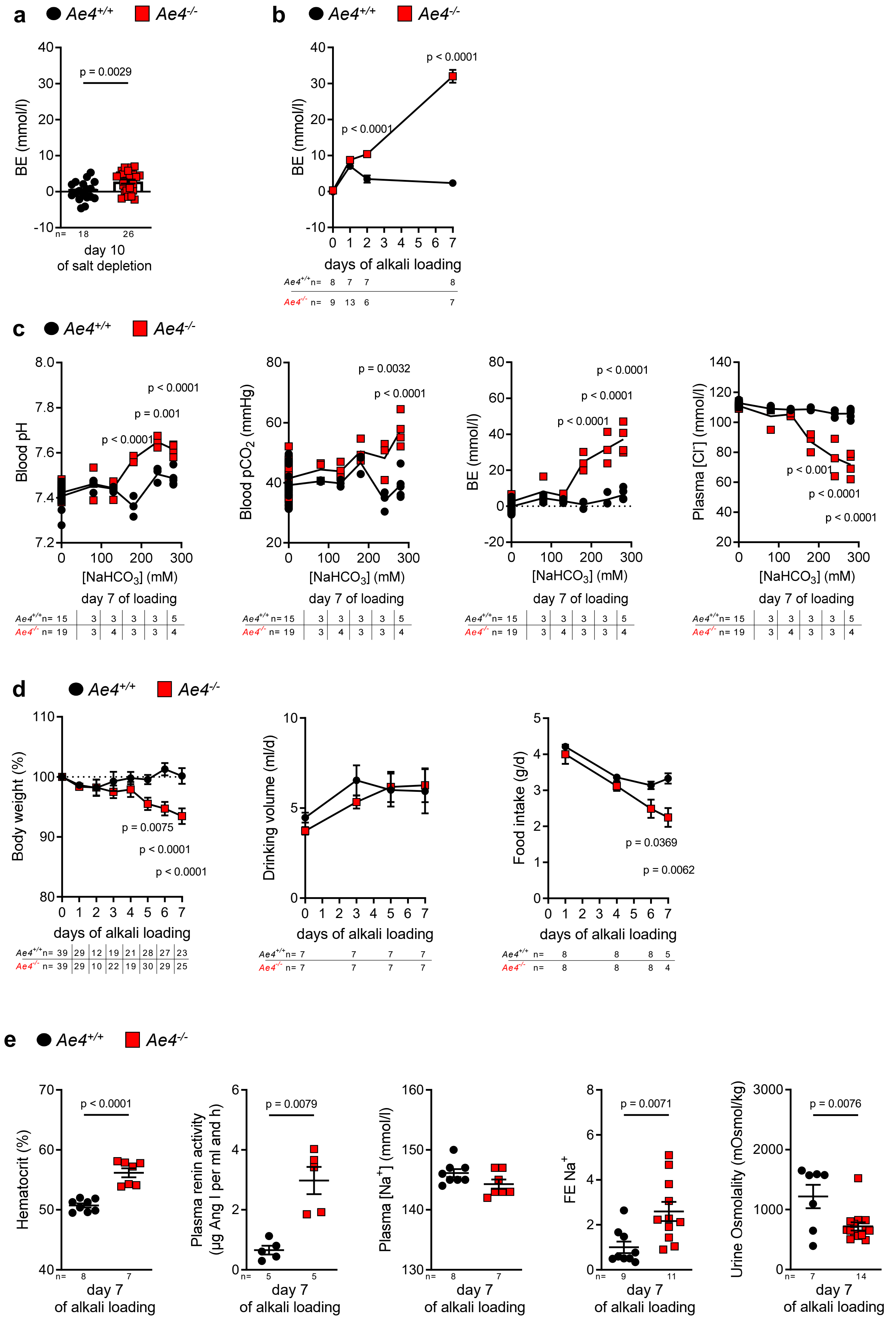
a
Ae4+/+
Ae4-/-
BE (mmol/l)
p = 0.0029
n= 18 26
day 10
of salt depletion
b
Ae4+/+
Ae4-/-
BE (mmol/l)
p < 0.0001
p < 0.0001
days of alkali loading
Ae4+/+ n= 8 7 7 8
Ae4-/- n= 9 13 6 7
c
Ae4+/+
Ae4-/-
Blood pH
p < 0.0001
p = 0.001
p < 0.0001
Blood pCO2 (mmHg)
p = 0.0032
p < 0.0001
BE (mmol/l)
p < 0.0001
p < 0.0001
p < 0.0001
Plasma [Cl-] (mmol/l)
p < 0.001
p < 0.0001
p < 0.0001
[NaHCO3] (mM)
day 7 of loading
Ae4+/+ n= 15 3 3 3 3 5
Ae4-/- n= 19 3 4 3 3 4
d
Ae4+/+
Ae4-/-
Body weight (%)
p = 0.0075
p < 0.0001
p < 0.0001
days of alkali loading
Ae4+/+ n= 39 29 12 19 21 28 27 23
Ae4-/- n= 39 29 10 22 19 30 29 25
Drinking volume (ml/d)
Ae4+/+ n= 7 7 7 7
Ae4-/- n= 7 7 7 7
Food intake (g/d)
p = 0.0369
p = 0.0062
Ae4+/+ n= 8 8 8 5
Ae4-/- n= 8 8 8 4
e
Ae4+/+
Ae4-/-
Hematocrit (%)
p < 0.0001
n= 8 7
Plasma renin activity (µg Ang I per ml and h)
p = 0.0079
n= 5 5
Plasma [Na+] (mmol/l)
n= 8 7
FE Na+
p = 0.0071
n= 9 11
Urine Osmolality (mOsmol/kg)
p = 0.0076
n= 7 14
day 7
of alkali loading

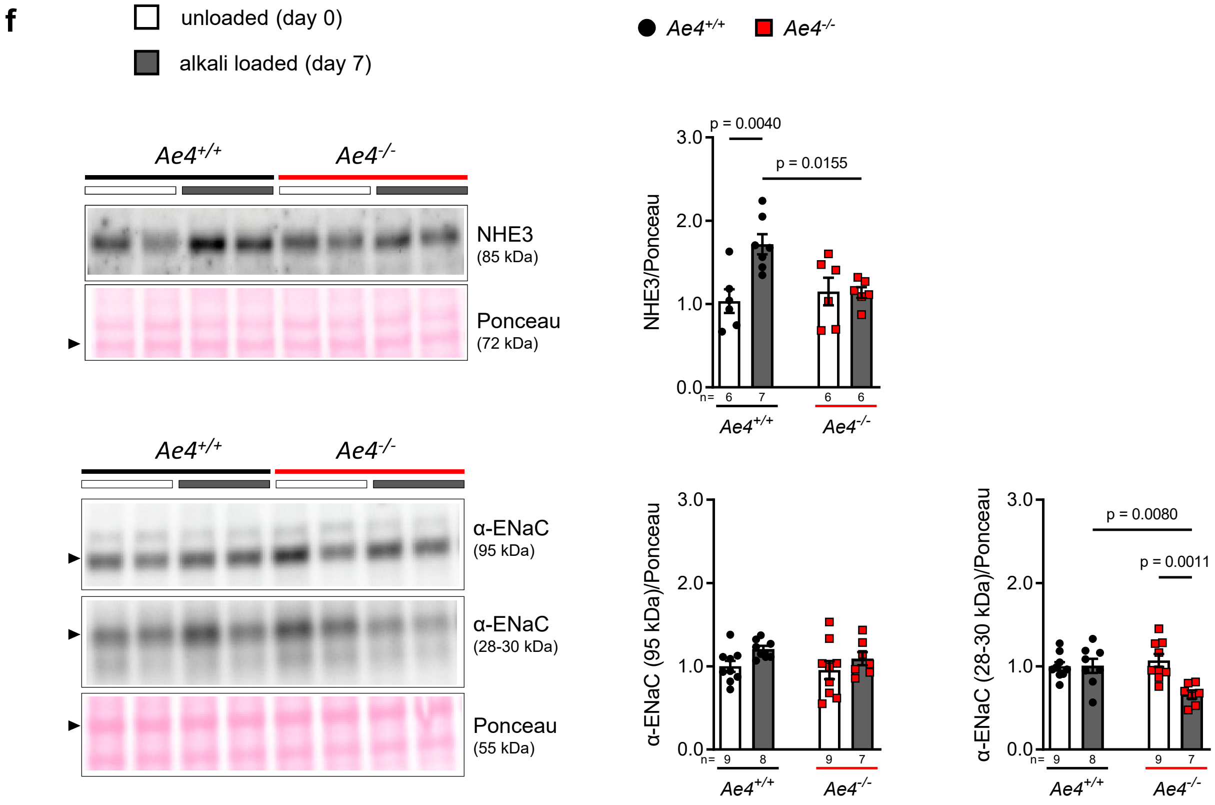

Supplementary Fig. 3 Altered acid base homeostasis and its impact on renal sodium and water handling in AE4-deficient mice.

a Base excess (BE) in the blood of *Ae4*$^{-/-}$ and *Ae4*$^{+/+}$ mice fed a salt-depleted diet for 10 days (n=18-26 animals per genotype, two-tailed Student's t-test). **b** *Ae4*$^{+/+}$ and *Ae4*$^{-/-}$ littermates were alkali-loaded for up to 7 days under salt depleted conditions (low salt diet and 230 mM $NaHCO_3$ added to the drinking water). Unloaded (day 0) mice received normal diet. Base excess (BE) in the blood of *Ae4*$^{-/-}$ mice aggravated over time (n=6-13 animals per genotype and day of alkali-loading, two-way ANOVA followed by Bonferroni's multiple comparisons test for *Ae4*$^{+/+}$ vs *Ae4*$^{-/-}$). **C** Dose-dependent effect of $NaHCO_3$ in the drinking water on acid-base status. Salt-depleted *Ae4*$^{+/+}$ and *Ae4*$^{-/-}$ mice were alkali loaded for 7 days with different concentrations of $NaHCO_3$ (0, 80, 130, 180, 240, or 280 mM) in the drinking water. The blood pH, pCO_2, BE, and plasma [Cl^-] of the mice are shown. Note that the severity of hypochloremic alkalosis in *Ae4*$^{-/-}$ mice is dose-dependent as hypochloremia and alkalosis increased with the amount of $NaHCO_3$ in the drinking water (n=3-19 animals each concentration and genotype, two-way ANOVA followed by Bonferroni's multiple comparisons test for *Ae4*$^{+/+}$ vs *Ae4*$^{-/-}$). **d-f** *Ae4*$^{+/+}$ and *Ae4*$^{-/-}$ littermates were alkali-loaded for up to 7 days under salt depleted conditions (low salt diet and 230 mM $NaHCO_3$ added to the drinking water). Unloaded (day 0) mice received normal diet. **d** Relative changes in body weight, drinking volume, and food intake over time. **e** After 7 days of alkali loading plasma [Na^+] was comparable, but blood hematocrit, plasma renin activity, and fractional excretion of sodium (FE Na^+) were higher in *Ae4*$^{-/-}$ than in *Ae4*$^{+/+}$ mice. Urine osmolality was lower in *Ae4*$^{-/-}$ than in *Ae4*$^{+/+}$ mice (n=5-14 animals per genotype, two-tailed Student's t-test). **f** Representative immunoblots of sodium-hydrogen-exchanger (NHE3) and epithelial sodium channel (α-ENaC) in *Ae4*$^{+/+}$ and *Ae4*$^{-/-}$ whole kidney lysates. Ponceau red staining served as control for loading. Graphs exhibit densitometric quantification of the protein levels (normalized to the levels found in *Ae4*$^{+/+}$ mice at day 0, n=5-9 animals each genotype and day of diet, one-way ANOVA followed by Bonferroni's multiple comparisons test). NHE3 and α-ENaC, which represent the major sodium reabsorption pathways in the proximal tubule and principal cells, resp., were reduced in *Ae4*$^{-/-}$ mice after alkali-loading compared to *Ae4*$^{+/+}$ littermates. All data are presented as mean ± SEM (n=animals). In **a, c, e, f** each point or square denotes one animal. Source data are provided as a Source Data file.

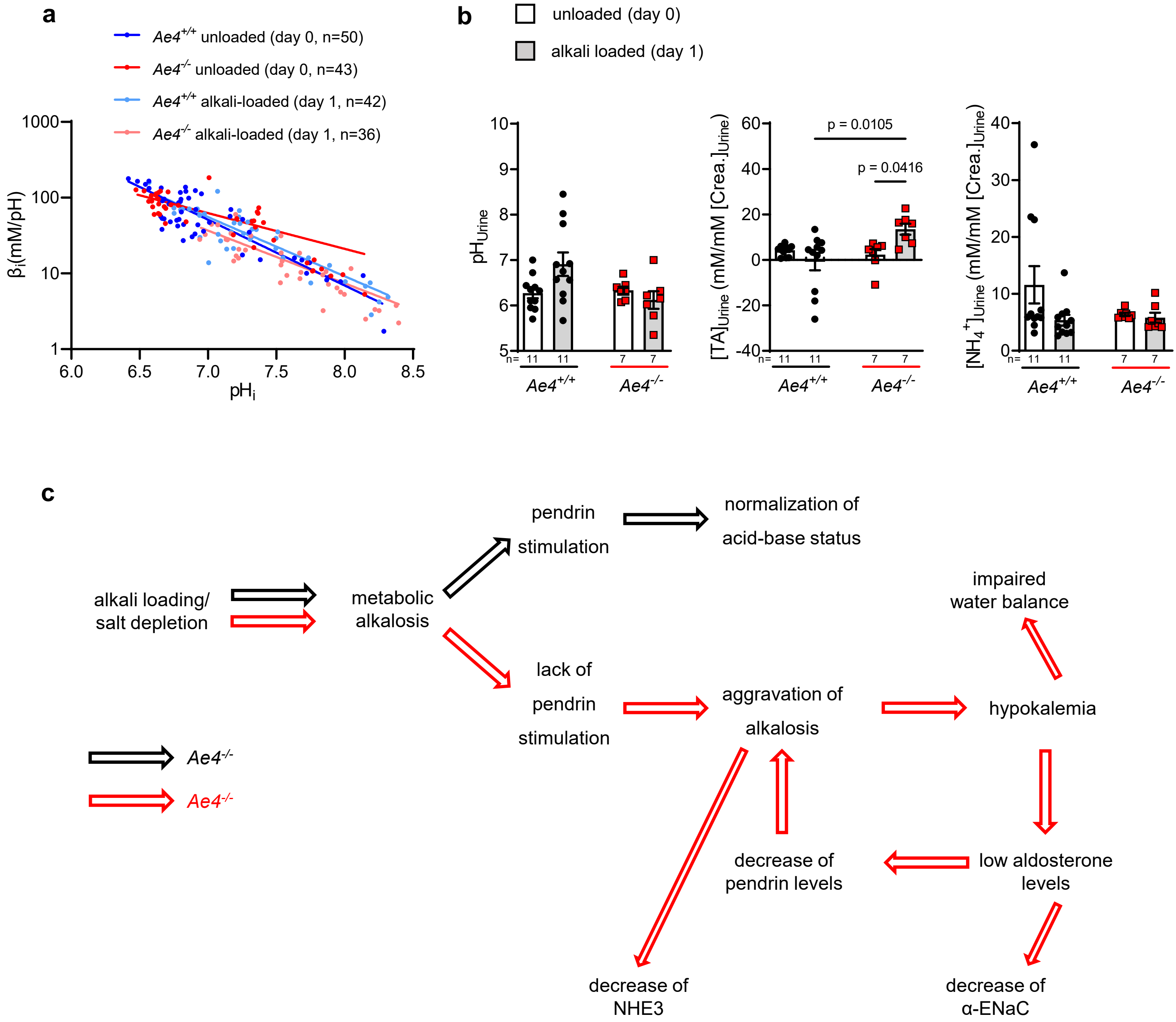

Supplementary Fig. 4 Insufficient pendrin activity in salt depleted *Ae4*$^{-/-}$ mice upon alkali loading. Wildtype (*Ae4*$^{+/+}$) and *Ae4*$^{-/-}$ littermates were alkali-loaded for up to 7 days under salt-depletion (low salt diet with 230 mM $NaHCO_3$ added to the drinking water). Before the alkali-loading mice received normal diet (day 0). **a** Intracellular buffer capacity in β-intercalated cells in unloaded (day 0) and alkali-loaded (day 1) mice. Buffer capacity was not different between *Ae4*$^{+/+}$ and *Ae4*$^{-/-}$ littermates. **b** Graphs show urine pH and urinary excretion of titratable acid (TA) and NH_4^+ (normalized to urinary [creatinine]) before and one day after alkali loading (data are presented as mean ± SEM, n=7-11 animals each genotype, each point or square denotes one animal, Kruskal-Wallis followed by Dunn's multiple comparisons test). Decrease of urinary acid excretion was absent in alkali-loaded *Ae4*$^{-/-}$ mice. **c** As *Ae4*$^{-/-}$ mice lack pendrin stimulation upon alkali loading, they enter a vicious cycle of alkalosis, hypokalemia, hypoaldosteronism, decrease of pendrin and further aggravation of alkalosis. The derailed acid-base balance also affects sodium and water balance by affecting NHE3, α-ENaC, and the ability to concentrate urine. Source data are provided as a Source Data file.

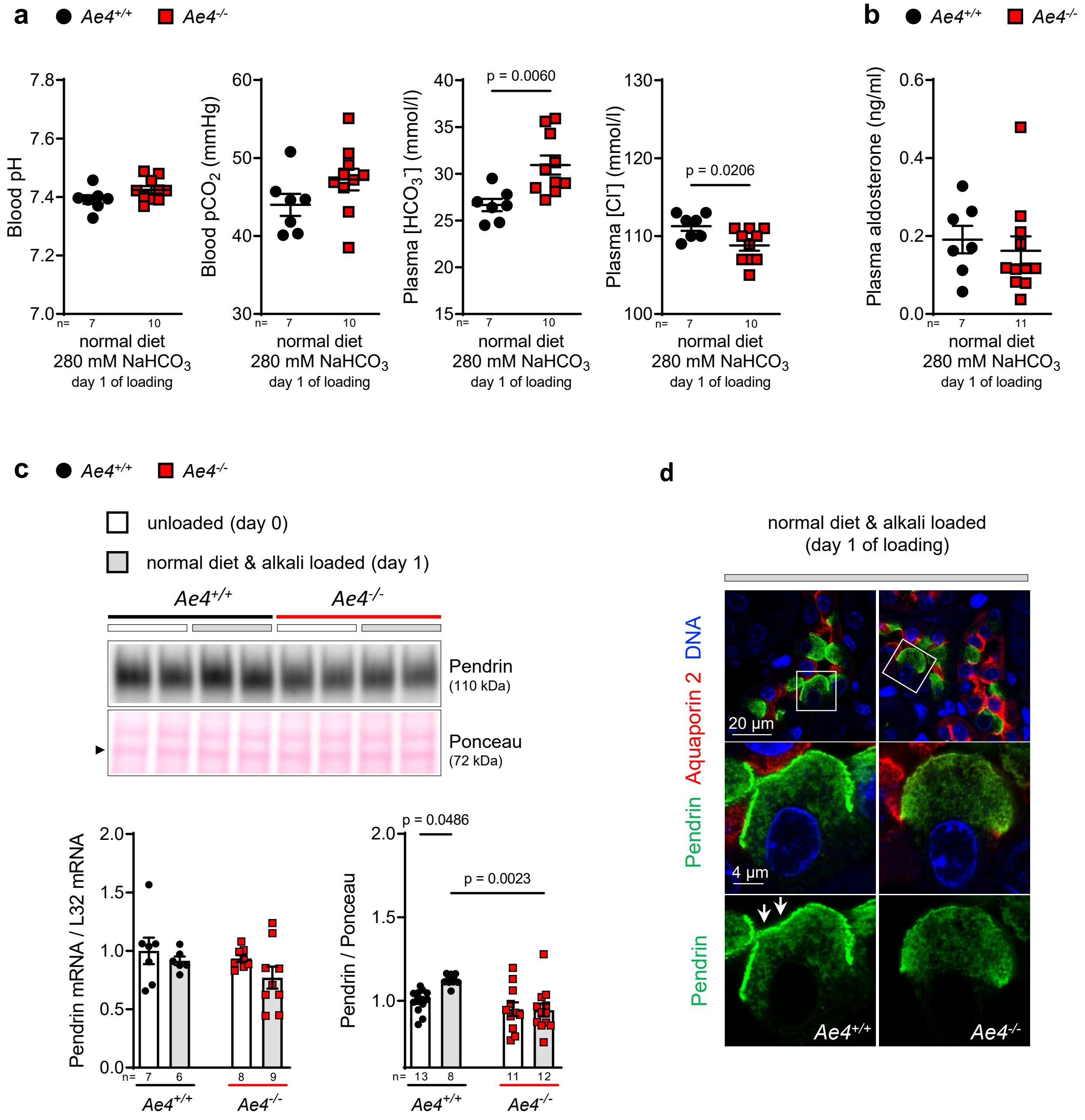

Supplementary Fig. 5 Insufficient activation of pendrin in *Ae4*$^{-/-}$ mice upon alkali loading is independent of the salt content of the diet. a-d Wildtype (*Ae4*$^{+/+}$) and *Ae4*$^{-/-}$ mice were alkali loaded for 1 day (normal salt diet with 280 mM $NaHCO_3$ added to the drinking water). Unloaded (day 0) mice received normal diet. **a** Graphs show blood pH, blood pCO_2, plasma [HCO_3^-], and plasma [Cl^-] of *Ae4*$^{+/+}$ and *Ae4*$^{-/-}$ littermates after 1 day of alkali loading (n=7-10 animals each genotype, two-tailed Student's t-test). **b** Plasma aldosterone levels of *Ae4*$^{+/+}$ and *Ae4*$^{-/-}$ littermates after 1 day of alkali loading (n=7-11 animals each genotype, two-tailed Student's t-test). **c** Representative immunoblots showing pendrin protein abundance in whole kidney lysates of *Ae4*$^{+/+}$ and *Ae4*$^{-/-}$ littermates before (day 0) and after 1 day of alkali loading. Ponceau red staining served as control for equal protein loading. Graphs exhibit densitometric quantification of mRNA und protein levels (normalized the levels found in unloaded *Ae4*$^{+/+}$ mice (day 0), n=6-13 animals each genotype, one-Way ANOVA followed by Bonferroni's multiple comparisons test). **d** Immunofluorescence staining of pendrin (green) and aquaporin 2 (red) in the kidneys of mice after 1 day of loading. In contrast to *Ae4*$^{+/+}$ mice (white arrows), no redistribution of pendrin to the apical membrane region upon alkali challenge was observed in *Ae4*$^{-/-}$ littermates. In **a-c** data are presented as mean ± SEM and each point or square denotes one animal. Source data are provided as a Source Data file.

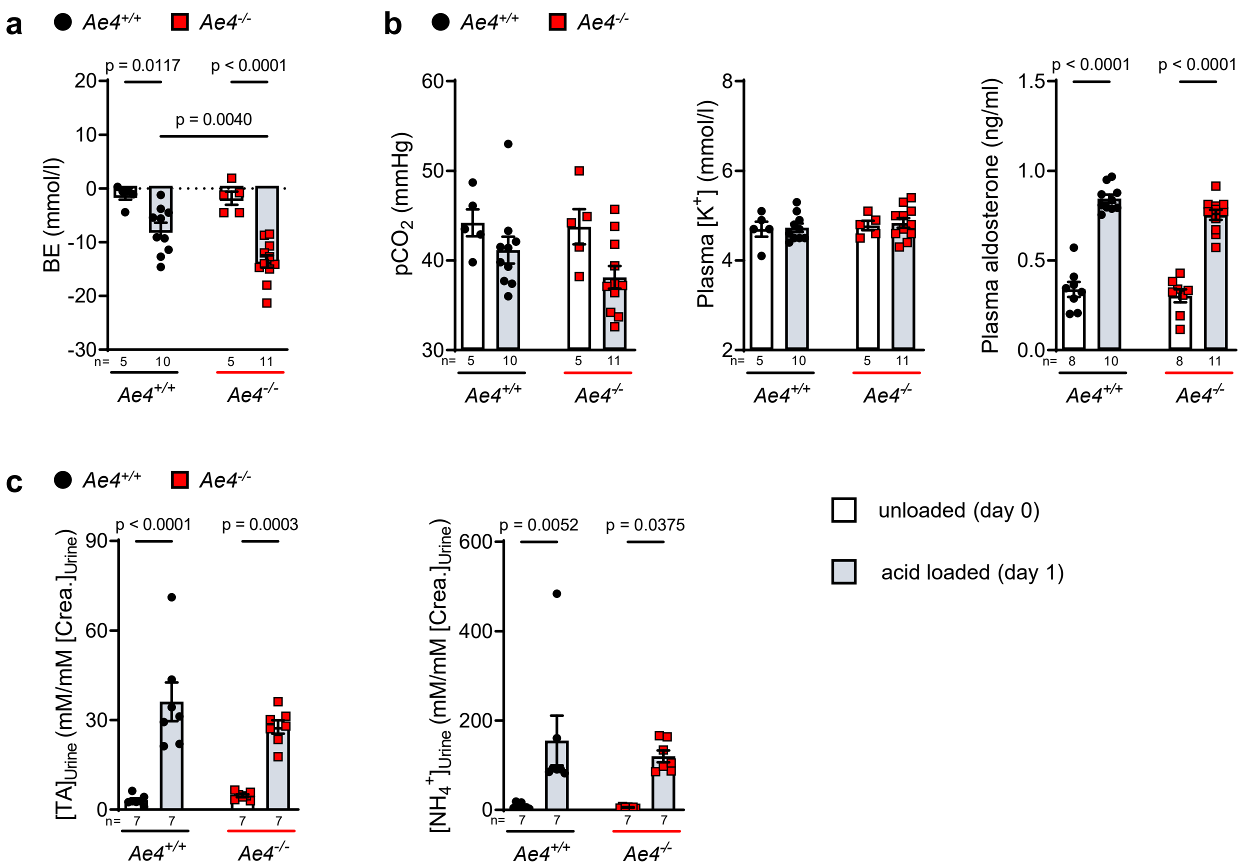

Supplementary Fig. 6 Systemic and renal responses to acid-loading during salt-depletion.
a-c Wildtype ($Ae4^{+/+}$) and $Ae4^{-/-}$ mice were acid loaded for 1 day under salt-depleted conditions (low salt diet with 280 mM NH_4Cl added to the sweetened drinking water). Unloaded (day 0) mice received normal diet. **a** Graph shows base excess (BE) of $Ae4^{+/+}$ and $Ae4^{-/-}$ littermates before (day 0) and after 1 day of loading (n=5-11 animals per genotype and treatment). **b** Graph shows blood pCO_2, plasma [K^+], and plasma aldosterone concentration in unloaded (day 0) and acid-loaded (day 1) mice (n=5-11 animals per genotype and treatment). **c** Urine acidification before and after 1 day of loading measured as urinary titratable acid ($[TA]_{Urine}$ normalized to the urinary [creatinine]) and as urinary $NH4^+$ ($[NH_4^+]_{Urine}$ normalized to urinary [creatinine], n=7 animals each genotype and treatment). All data are presented as mean ± SEM and each point or square denotes one animal. In **a-c** the significances were determined by one-way ANOVA followed by Bonferroni's multiple comparisons test. Source data are provided as a Source Data file.

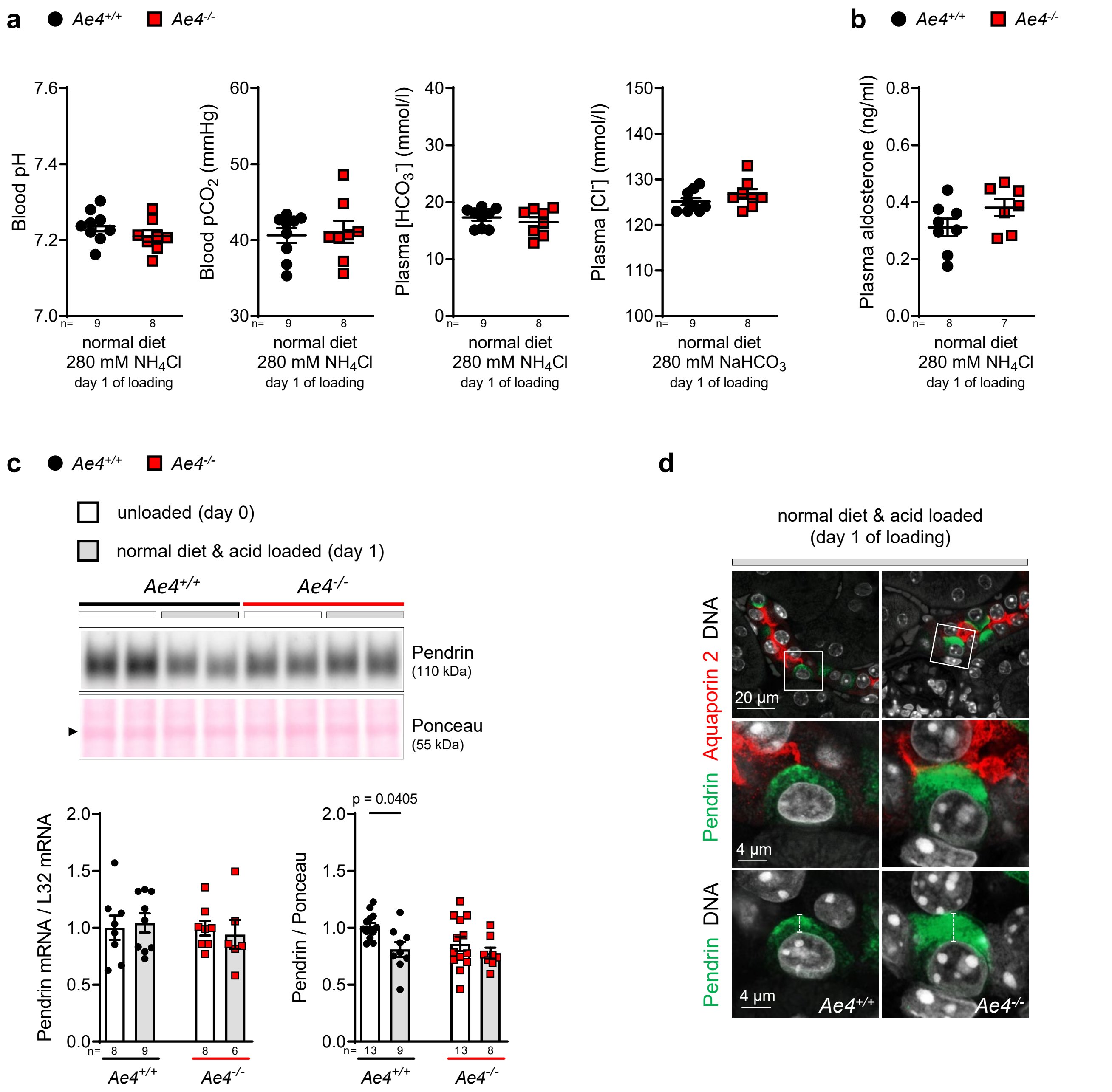

Supplementary Fig. 7 Systemic and renal response on day 1 of acid loading during normal salt intake. a-d Wildtype (*Ae4*$^{+/+}$) and *Ae4*$^{-/-}$ mice were acid loaded for 1 day (normal salt diet with 280 mM NH_4Cl added to the sweetened drinking water). Unloaded (day 0) mice received normal diet. **a** Graphs show blood pH, blood pCO_2, plasma [HCO_3^-], and plasma [Cl^-] of *Ae4*$^{+/+}$ and *Ae4*$^{-/-}$ littermates after 1 day of acid loading (n=8-9 animals each genotype, two-tailed Student's t-test). **b** Plasma aldosterone levels of *Ae4*$^{+/+}$ and *Ae4*$^{-/-}$ littermates after 1 day of acid loading (n=7-8 animals each genotype, two-tailed Student's t-test). **c** Representative immunoblots showing pendrin protein abundance in whole kidney lysates of *Ae4*$^{+/+}$ and *Ae4*$^{-/-}$ littermates before (day 0) and after 1 day of acid loading. Ponceau red staining served as control for equal protein loading. Graphs exhibit densitometric quantification of mRNA und protein levels (normalized the levels found in unloaded *Ae4*$^{+/+}$ mice (day 0), n=6-13 animals each genotype, one-way ANOVA followed by Bonferroni's multiple comparisons test). **d** Immunofluorescence staining of pendrin (green) and aquaporin 2 (red) in the kidneys of mice after 1 day of loading. In contrast to *Ae4*$^{+/+}$ mice (white lines), no reduction of pendrin cap size upon acid loading was observed in *Ae4*$^{-/-}$ littermates. In **a-c** data are presented as mean ± SEM and each point or square denotes one animal. Source data are provided as a Source Data file.

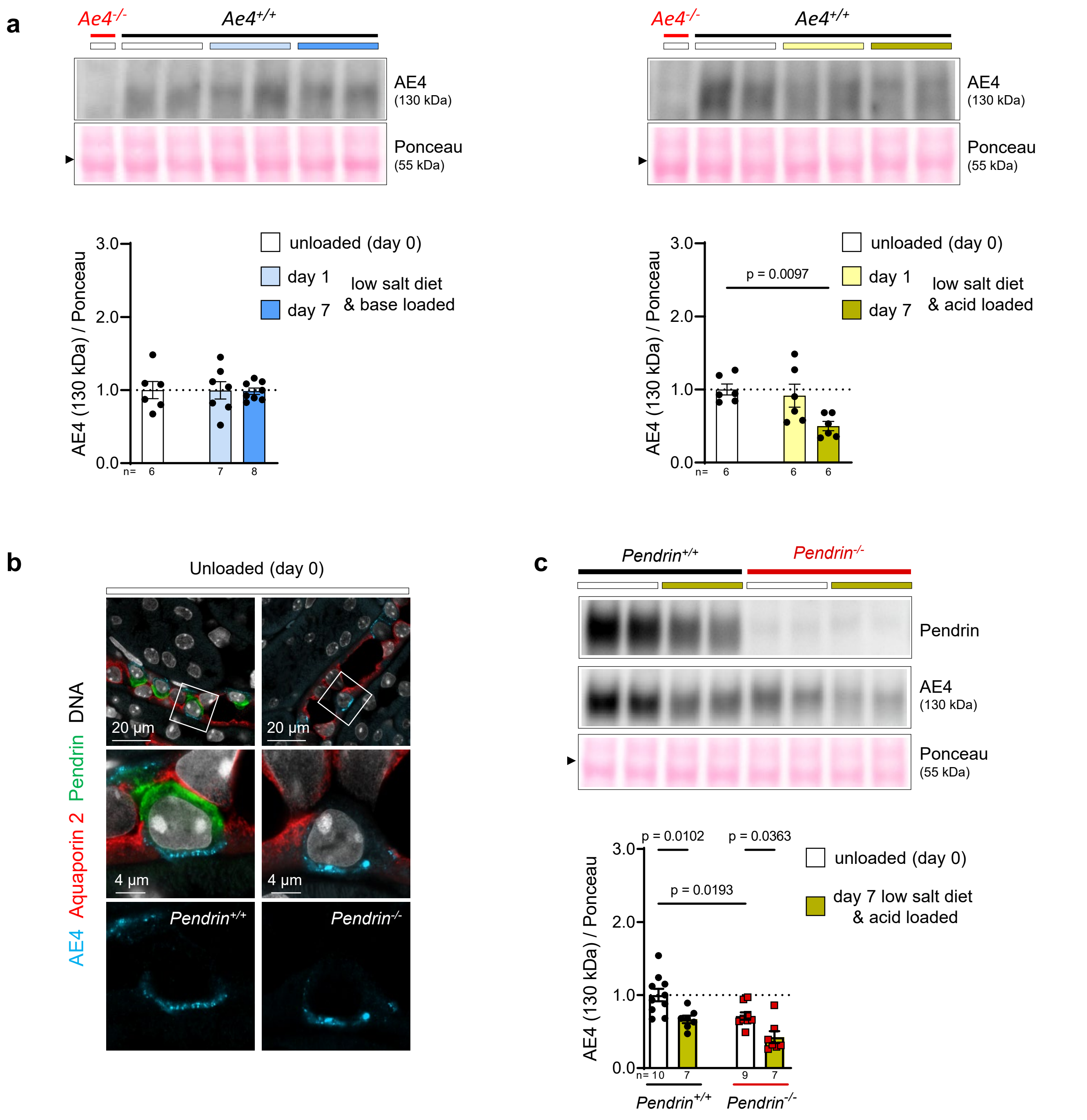

Supplementary Fig. 8 Regulation of AE4 during base and acid loading.
a Ae4 wildtype (*Ae4*$^{+/+}$) mice were base loaded (low salt diet with 230 mM $NaHCO_3$ in the drinking water) or acid loaded (low salt diet with 280 mM NH_4Cl in sweetened drinking water) for up to 7 days. Unloaded (day 0) mice received a normal diet. Representative immunoblots showing AE4 protein abundance in whole kidney lysates of *Ae4*$^{+/+}$ mice before (day 0) and after base or acid loading. *Ae4*$^{-/-}$ probe served as negative control and Ponceau red staining served as control for equal protein loading. Graphs exhibit densitometric quantification of protein levels (normalized to the levels found in unloaded *Ae4*$^{+/+}$ mice on day 0, n=6-8 animals each loading protocol). **b** Immunofluorescence staining of AE4 (turquoise), AQP2 (red), and pendrin (green) in the kidneys of unloaded Pendrin wildtype (*Pendrin*$^{+/+}$) and knockout (*Pendrin*$^{-/-}$) mice. The basolateral localization of AE4 was not affected by the lack of pendrin. **c** Pendrin wildtype (*Pendrin*$^{+/+}$) and knockout (*Pendrin*$^{-/-}$) mice were acid loaded (low salt diet with 280 mM NH_4Cl in sweetened drinking water) for 7 days. Unloaded (day 0) mice received a normal diet. Representative immunoblots showing pendrin and AE4 protein abundance in whole kidney lysates of the mice before (day 0) and after acid loading. Ponceau red staining served as control for equal protein loading. Graphs exhibit densitometric quantification of AE4 protein levels (normalized to the levels found in unloaded *Pendrin*$^{+/+}$ mice (day 0), n=7-10 animal each genotype and loading protocol). In **a, c** data are presented as mean ± SEM and each point or square denotes one animal. In **a, c** significance was determined by one-way ANOVA followed by Bonferroni's multiple comparisons test. Source data are provided as a Source Data file.
