# Supplementary material for: The AE4 transporter mediates kidney acid-base sensing
Source: Nat Commun. 2023 May 26;14:3051. doi: 10.1038/s41467-023-38562-x (PMC10220024; doi:10.1038/s41467-023-38562-x)
Supplement: Supplementary file 3 — Reporting Summary [file 41467_2023_38562_MOESM3_ESM.pdf]

nature portfolio

Corresponding author(s): Heimo Ehmke

Last updated by author(s): May 4, 2023

# Reporting Summary

Nature Portfolio wishes to improve the reproducibility of the work that we publish. This form provides structure for consistency and transparency in reporting. For further information on Nature Portfolio policies, see our Editorial Policies and the Editorial Policy Checklist.

## Statistics

For all statistical analyses, confirm that the following items are present in the figure legend, table legend, main text, or Methods section.

| n/a | Confirmed | |
|---|---|---|
| ☐ | ☒ | The exact sample size (*n*) for each experimental group/condition, given as a discrete number and unit of measurement |
| ☐ | ☒ | A statement on whether measurements were taken from distinct samples or whether the same sample was measured repeatedly |
| ☐ | ☒ | The statistical test(s) used AND whether they are one- or two-sided<br>*Only common tests should be described solely by name; describe more complex techniques in the Methods section.* |
| ☒ | ☐ | A description of all covariates tested |
| ☐ | ☒ | A description of any assumptions or corrections, such as tests of normality and adjustment for multiple comparisons |
| ☐ | ☒ | A full description of the statistical parameters including central tendency (e.g. means) or other basic estimates (e.g. regression coefficient) AND variation (e.g. standard deviation) or associated estimates of uncertainty (e.g. confidence intervals) |
| ☐ | ☒ | For null hypothesis testing, the test statistic (e.g. *F*, *t*, *r*) with confidence intervals, effect sizes, degrees of freedom and *P* value noted<br>*Give P values as exact values whenever suitable.* |
| ☒ | ☐ | For Bayesian analysis, information on the choice of priors and Markov chain Monte Carlo settings |
| ☒ | ☐ | For hierarchical and complex designs, identification of the appropriate level for tests and full reporting of outcomes |
| ☒ | ☐ | Estimates of effect sizes (e.g. Cohen's *d*, Pearson's *r*), indicating how they were calculated |

*Our web collection on statistics for biologists contains articles on many of the points above.*

## Software and code

Policy information about availability of computer code

| | |
|---|---|
| Data collection | LSM800 with Airyscan confocal microscope with ZenBlue v3.6 and ZenBlack software v3.0 (Zeiss), Multiskan FC with Skanit Software v3.1 (Thermo Fisher Scientific), ABL 90 series blood gas analyzer (Radiometer), Quant Studio 5 Real-Time-PCR-System v1.3 (Applied Biosystems, Thermo Fisher Scientific), Fusion FX7 EDGE v0.7 Imager (Vilbert Lourmat). |
| Data analysis | GraphPad Prism v9.5.1, Adobe Photoshop Version 23.0.1, Excel 2016, Image J v2.0 (FIJI), Bio 1D software v15.08b (Vilbert Lourmat), Quant Studio 5 Real-Time-PCR-System design & analysis software v1.5.1 (Applied Biosystems, Thermo Fisher Scientific) |

For manuscripts utilizing custom algorithms or software that are central to the research but not yet described in published literature, software must be made available to editors and reviewers. We strongly encourage code deposition in a community repository (e.g. GitHub). See the Nature Portfolio guidelines for submitting code & software for further information.

## Data

Policy information about availability of data

All manuscripts must include a data availability statement. This statement should provide the following information, where applicable:

- Accession codes, unique identifiers, or web links for publicly available datasets
- A description of any restrictions on data availability
- For clinical datasets or third party data, please ensure that the statement adheres to our policy

All raw data are available within the paper, its supplementary information files (including the source data file).

## Human research participants

Policy information about studies involving human research participants and Sex and Gender in Research.

| | |
|---|---|
| Reporting on sex and gender | The human kidney was removed in the setting of a tumor resection, the healthy (tumor free) part of kidney not needed for pathological diagnosis was used. Patients provided written consent for the use of samples for research. Because patients were anonymized, information on age and gender are not available. |
| Population characteristics | n/a |
| Recruitment | n/a |
| Ethics oversight | Sample collection was not done by the investigators and all data are anonymized; therefore, they are exempt from IRB approval (§12 HambKHG). |

Note that full information on the approval of the study protocol must also be provided in the manuscript.

# Field-specific reporting

Please select the one below that is the best fit for your research. If you are not sure, read the appropriate sections before making your selection.

[x] Life sciences  [ ] Behavioural & social sciences  [ ] Ecological, evolutionary & environmental sciences

For a reference copy of the document with all sections, see nature.com/documents/nr-reporting-summary-flat.pdf

# Life sciences study design

All studies must disclose on these points even when the disclosure is negative.

| | |
|---|---|
| Sample size | All experiments reported in this study were independently repeated at least three times. Representativ confocal images were derived from at least three differrent mice per diet and genotype. qRT-PCR data were performed in duplicate. Each data point provided within the paper and source data file is from a single mouse, except in Fig. 2D (three mice per genotype and diet), Fig. 2E and Fig. S4A (n = number of ß-cells).<br>Urinary excretion data or food and water intake data in Fig. 1C, Fig. 2F, Fig. 3E, Fig. 3F, Fig. S1A, Fig. S2A, Fig. S3D, Fig. S4B, and Fig. S6C were obtained at different time points in individual mice (paired experiments).<br>In addition sample size was calculated a priori with GPower. |
| Data exclusions | RT-qPCR: some data points were excluded due to technical issues (unspecific melting curves). |
| Replication | All experiments reported in this study were independently repeated at least three times, while all attempts at replication were successful. |
| Randomization | Wildtype and knockout mice were allocated to specific groups based on their genotype. Further allocation to the different treatment groups was randomized. For across-species analysis of AE4 expression (Fig. 1A) there was no allocation into experimental groups. All other experiments involved mice. |
| Blinding | The investigators were blinded to group allocation during data collection and analysis. |

# Reporting for specific materials, systems and methods

We require information from authors about some types of materials, experimental systems and methods used in many studies. Here, indicate whether each material, system or method listed is relevant to your study. If you are not sure if a list item applies to your research, read the appropriate section before selecting a response.

### Materials & experimental systems

| n/a | Involved in the study | |
|---|---|---|
| [ ] | [x] | Antibodies |
| [x] | [ ] | Eukaryotic cell lines |
| [x] | [ ] | Palaeontology and archaeology |
| [ ] | [x] | Animals and other organisms |
| [x] | [ ] | Clinical data |
| [x] | [ ] | Dual use research of concern |

### Methods

| n/a | Involved in the study | |
|---|---|---|
| [x] | [ ] | ChIP-seq |
| [x] | [ ] | Flow cytometry |
| [x] | [ ] | MRI-based neuroimaging |

## Antibodies

| | |
|---|---|
| Antibodies used | rabbit anti-AE4 (Alpha diagnostics AE41-A IF1:200, WB: 1:1000), goat anti-AQP-2 (Santa Cruz sc-9882), guinea pig anti-pendrin (C.A. Wagner Lab), rabbit anti-pendrin (C.A. Wagner Lab), rabbit anti-α-ENaC (J. Loffing Lab, Zürich), rabbit anti-β-ENaC (StressMarq SPC 404), rabbit anti γ-ENaC (StressMarq SPC 405), rabbit anti-NCC (Millipore AB3553), sheep anti-pNCCThr60 (MRC PPU S995B), and rabbit anti-NHE3 (StressMarq SPC 400D) |
| Validation | We thouroughly describe the protocol for the usage of antibodies in our manuscript. All commercially available antibodies (anti-AQP-2, anti-β-ENaC, anti γ-ENaC, anti-NCC, anti-pNCCThr60, and anti-NHE3 were validated by the respective manufacturer (as indicated in the specific data sheets) or publication validated. The AE4 antibody was validated by us. We used Ae4 knockout mice to check the specificity of the AE4 antibody for both immunofluorescence and western blot (shown in supplementary figure 8). Pendrin antibodies have been previously used by CA Wagner (Hafner P. et al. Am J Physiol Cell Physiol 295, C1658-1667, doi:10.1152/ajpcell.00419.2008) and ENaC antibody have been used by J. Loffing (Sorensen MV Kidney Int 83, 811-824, doi:10.1038/ki.2013.14 (2013). For quality control, we titrate each antibody to determine its dilution for optimal performance for each application. |

## Animals and other research organisms

Policy information about studies involving animals; ARRIVE guidelines recommended for reporting animal research, and Sex and Gender in Research

| | |
|---|---|
| Laboratory animals | Slc4a9 knockout and wildtype male mice were obtained from the animal facility of University Medical Center Hamburg-Eppendorf and transferred to the institutional animal facility at least 2 weeks before the experiments. Chow and water provided ad libitum. Mice were maintained in a normal 12-hour light/ 12-hour dark cycle at a room temperatur from 20°C to 24°C and controlled humidity. Experiments were performed using 8 - 16 week-old males. Male and female Slc26a4 knockout and wildtype mice (JAX stock #01842432) were obtained from the animal facility of Aarhus University and were transferred to the institutional animal facility at least 2 weeks before the experiments. Experiments were performed using 10 - 29 week-old mice. |
| Wild animals | Our study did not involve wild animals. |
| Reporting on sex | Slc4a9 only male mice.<br>Slc26a4 male and female mice |
| Field-collected samples | Our study did not involve field-collected sample. |
| Ethics oversight | All procedures were approved by the University Medical Center Hamburg-Eppendorf institutional guidelines and institutional animal welfare officer. The procedures were conform to the requirements of the German Animal Welfare Act. Approvals obtained from the State Authority of Hamburg (Freie und Hansestadt Hamburg, Behörde für Justiz und Verbraucherschutz, Lebensmittelsicherheit und Veterinärwesen), Germany (G91/14 and N108/2019). |

Note that full information on the approval of the study protocol must also be provided in the manuscript.
